# Artificial Crown and Bridge Work

**Published:** 1889-10

**Authors:** 


### Bibliographical.

ARTIFICIAL CROWN AND BRIDGE WORK.—A practical treatise, by George Evans. Published by the S. S. White Dental Manufacturing Co., Philadelphia, 1888.

Part 1. Is devoted to the preparatory treatment of roots and teeth, and describes methods of preservation and devitalization of pulps of teeth, treatment of abscess, and shaping of teeth and roots for crown-work.

Part 2. Describes the Bonwill, Howe, Gates, Foster, Howland, Logan, Weston, Brown and new Richmond porcelain crowns, and the gold collar and vulcanite attachments, porcelain fronts, partial crowns, crowning of fractured teeth and roots and irregular teeth.

Part 3. Is devoted to bridge-work, such as extension, double bar, detachable and removable bridges, and repair of crown or bridge work.

Part 4. Describes the materials and processes used in crown and bridge work, such as plate and solder, porcelain teeth, molds and dies, soldering instruments and appliances.

All of which methods are illustrated by many excellent cuts and comprehensive descriptions, rendering the work a valuable addition to the text books of our profession.

